# Days spent on non-invasive ventilation support: can it determine when to initiate VV- ECMO? Observational study in a cohort of Covid-19 patients

**DOI:** 10.1186/s12890-023-02605-2

**Published:** 2023-08-26

**Authors:** María P. Fuset-Cabanes, LLuisa Hernández-Platero, Joan Sabater-Riera, Miguel Gordillo-Benitez, Fabio Di Paolo, Paola Cárdenas-Campos, Krystel Maisterra-Santos, María Pons-Serra, Paola Sastre-Pérez, Alejandro García-Zaloña, Javiera Puentes-Yañez, Xosé Pérez-Fernández

**Affiliations:** 1 Critical Care Medicine, Hospital Universitari de Bellvitge, Barcelona, Spain; 2 Pediatric Intensive Care Unit, SJD Barcelona Hospital, Barcelona, Spain

**Keywords:** ARDS, Extracorporeal membrane oxygenation, Non-invasive ventilation, COVID-19, Respiratory insufficiency

## Abstract

**Background:**

The study evaluates the impact of the time between commencing non-invasive ventilation (NIV) support and initiation of venovenous extracorporeal membrane oxygenation (VV-ECMO) in a cohort of critically ill patients with coronavirus disease 2019 (COVID-19) associated acute respiratory distress syndrome (ARDS).

**Methods:**

Prospective observational study design in an intensive Care Unit (ICU) of a tertiary hospital in Barcelona (Spain). All patients requiring VV-ECMO support due to COVID-19 associated ARDS between March 2020 and January 2022 were analysed. Survival outcome was determined at 90 days after VV-ECMO initiation. Demographic data, comorbidities at ICU admission, RESP (respiratory ECMO survival prediction) score, antiviral and immunomodulatory treatments received, inflammatory biomarkers, the need for vasopressors, the thromboprophylaxis regimen received, and respiratory parameters including the length of intubation previous to ECMO and the length of each NIV support (high-flow nasal cannula, continuous positive airway pressure and bi-level positive airway pressure), were also collated in order to assess risk factors for day-90 mortality. The effect of the time lapse between NIV support and VV-ECMO on survival was evaluated using logistic regression and adjusting the association with all factors that were significant in the univariate analysis.

**Results:**

Seventy-two patients finally received VV-ECMO support. At 90 days after commencing VV-ECMO 35 patients (48%) had died and 37 patients (52%) were alive. Multivariable analysis showed that at VV-ECMO initiation, age (*p* = 0.02), lactate (*p* = 0.001), and days from initiation of NIV support to starting VV-ECMO (*p* = 0.04) were all associated with day-90 mortality.

**Conclusions:**

In our small cohort of VV-ECMO patients with COVID-19 associated ARDS, the time spent between initiation of NIV support and VV-ECMO (together with age and lactate) appeared to be a better predictor of mortality than the time between intubation and VV-ECMO.

## Background

The number of days spent on mechanical ventilation before interventions, such as venovenous extracorporeal membrane oxygenation (VV-ECMO) [1] or steroids [2], has been used to classify different clinical stages of the acute respiratory distress syndrome (ARDS). This classification tries to differentiate between an early phase, most of the times defined within the first week from endotracheal intubation (ETI) when interventions could potentially change the ARDS evolution, and a late phase, normally more than seven days from ETI when interventions would theoretically be ineffective. This classification, where *day 0* is defined by the day of ETI, represents a somewhat historic cohort given that in the past most of the patients with hypoxemic acute respiratory failure (ARF) were not treated with any form of non-invasive ventilation (NIV) prior to ETI.

During the coronavirus disease 2019 (COVID-19) pandemic, the use of NIV for hypoxemic ARF extraordinarily increased, initially due to logistical reasons [3], but soon after it became a more standard practice with observational reports showing that this NIV approach could be associated with a decrease in mortality (although study designs do not allow inferring causality) especially when compared to that of an early intubation approach [4–6]. NIV in whatever form it be, high-flow nasal cannula (HFNC), continuous positive airway pressure (CPAP), or bi-level positive airway pressure (BiPAP), probably heralds the initial stage of alveolar collapse of ARDS where positive pressure is required to maintain adequate oxygenation [7, 8]. HFNC can be included as a NIV modality but important differences exist in the level of positive pressure that can provide support to reduce the work of breathing and improve oxygenation. Furthermore, important concerns still exist respect to the effect that these pressures may have in the lung specially regarding the patient’s self-inflicted lung injury (P-SILI) hypothesis [9] although no solid scientific data has demonstrated so far that invasive ventilation would be less harmful than NIV, especially when high PEEP is required or recruitment manoeuvres are performed [10, 11].

Of note, VV-ECMO has been extensively used during the pandemic, but some authors report that VV-ECMO outcomes appear to have worsened in parallel with an increased use of NIV [12]. This has been explained, in part, through the hypothesis of a P-SILI mediated effect [11], although ARDS severity (clinical stage) might be another reason as these patients receiving previous NIV support would probably be initiated on ECMO later than those who are directly intubated. Regardless of this last hypothesis and of previous publications, no data to-date has been reported regarding the number of days VV-ECMO patients spend on NIV support that may influence their final outcome.

Our hypothesis is that the time lapse between NIV initiation and VV-ECMO is as important as the time lapse between ETI and VV-ECMO when considering initiating a patient on VV-ECMO due to hypoxemic ARF. To demonstrate this hypothesis, we studied all patients requiring VV-ECMO during the COVID-19 pandemics and we evaluated all mortality risk factors with special consideration to all those factors related to the use of NIV previous to VV-ECMO. The aim in this single centre study was to examine the relationship between the use of NIV and mortality in VV-ECMO patients during the COVID-19 pandemic and whether this could be correlated with the time spent on NIV rather than to the use of NIV, per se.

## Methods

We collected data prospectively from our intensive care unit (ICU) of our university hospital (Hospital Universitari de Bellvitge, Barcelona) in Spain. We included all adults with ARDS (according to Berlin definition) [13] who required VV-ECMO and SARS-Cov-2 infection as tested by real-time reverse transcriptase-polymerase chain reaction in respiratory fluids, between March 2020 to January 2022.

All demographic data, relevant comorbidities, and Sequential Organ Failure Assessment (SOFA) score at ICU admission were collected. SOFA and RESP (respiratory ECMO survival prediction) scores were noted on initiation of VV-ECMO. Antiviral and immunomodulatory treatments received, the need for vasopressors (yes/no), the thromboprophylaxis regimen received, and respiratory parameters including the length of each ventilation support were also collected. Criteria for ETI and VV-ECMO initiation followed the recommendations from the Spanish critical care society (SEMICYUC) [14] and ELSO respectively [15] although application of these criteria did depend on our hospital crisis capacity, especially during the first three months of pandemic (March–May, 2020). Survival at 90 days after VV-ECMO was monitored and initiation of NIV was considered when patients started on any available modality (HFNC, CPAP, or BiPAP). In order to evaluate the global effect of the time lapse between NIV and VV-ECMO, we had to make the presumption that in those patients who did not receive NIV, their day 0 was the day of intubation. Univariate analyses were conducted to explore the association between death at 90 days after VV-ECMO initiation and each of the predefined risk factors. Continuous variables were compared using sample t-test and Mann Whitney test. Categorical data were compared using the Chi-square test or Fisher exact test. The effect of the time lapse between NIV and VV-ECMO on survival was evaluated using logistic regression and adjusting the association with all factors that were significant in the univariate analysis. The association measures were calculated (adjusted odds ratio [OR]) with a confidence interval (CI) of 95%. The study was approved by the medical ethics committee at Bellvitge Hospital (PR40/21).

## Results

**Fig. 1:**
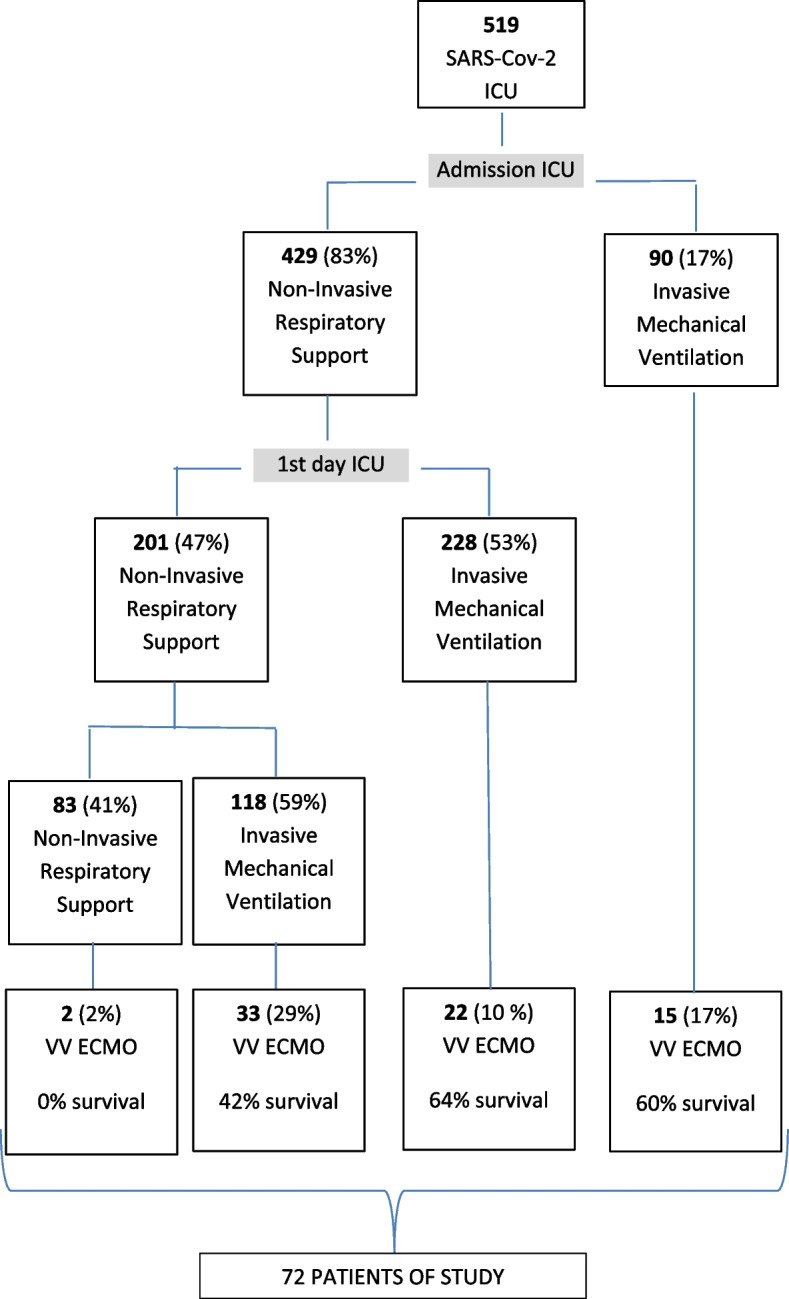
Flowchart

From March 2020 to January 2022, 519 subjects with SARS-Cov-2 and ARDS criteria were admitted to the ICU. 429 patients (83%) had received some modality of NIV before ICU admission with a median (IQR) time of 1 (0–3) days. Within the first day from ICU admission 318 patients (61%) had been intubated but the other 201 (39%) subjects continued on NIV support from which 118 (59%) were finally intubated. Among these ARDS patients, for the purpose of our study we only evaluated the 72 subjects (14%) that were finally initiated on VV-ECMO (Fig. 1).

The median (IQR) time from intubation to VV-ECMO was 4 (2-9) days whereas the complete median time spent from NIV support to ECMO (which also includes the time spent with intubation) was 9 (5-15) days. Table 1, shows the demographic data, clinical characteristics and outcomes of these 72 patients according to their survival status 90 days after VV-ECMO initiation. As demonstrated 35 patients (48%) had died and 37 patients (52%) were alive. Patients who were alive at 90 days after VV-ECMO initiation were younger and had a shorter time lapse from NIV to VV-ECMO.

**Table 1:** Characteristics of Patients with COVID-19 and ARDS requiring ECMO

Characteristics	All patients	ECMO survivors	ECMO nonsurvivors	p
	(*n* = 72)	*n* = 37 (52%)	*n* = 35 (48%)	
Age, yr, mean (SD)	53 (8)	50.4 (9)	55.8 (7)	**0.01**
Sex, M, n (%)	58 (80.5)	29 (80.5)	28 (77.8)	1
Body mass index, kg/m^2^,mean (SD)	31.8 (6)	32.0 (6)	31.5 (6)	0.7
SOFA at ICU admission, median (IQR)	7 (5 -9)	6.5 (4—8)	7 (4—8)	0.9
Pandemic waves, n (%)				0.6
First (March–May 2020)	15 (20.8)	10 (60)	5 (40)	
Rest	57 (79.2)	27 (47.4)	30 (52.6)	
Pre-ECMO comorbidities, n (%)				
Hypertension	29 (40.3)	13 (35.1)	16 (45.7)	0.8
Diabetes mellitus	16 (22.2)	7 (18.9)	9 (25.7)	0.9
Chronic pulmonary disease	5 (6.9)	0 (0)	5 (14.3)	0.2
Pre-ECMO treatments, n (%)				
Prono positioning	66 (92)	34 (92)	32 (91)	0.7
Neuromuscular blockade	67 (45)	33 (89)	34 (97)	1
Inhaled nitric oxide	33 (46)	15 (41)	18 (51)	0.6
Corticosteroids	65 (91.5)	34 (92)	31 (89)	0.4
Tocilizumab	30 (41.7)	15 (41)	15 (43)	1
Anticoagulation	19 (26.7)	14 (38)	5 (14)	0.2
Vasopressors	21 (29)	9 (24)	12 (34)	0.4
Pre-ECMO ventilatory support, n (%)				
HFNC	53 (73.6)	24 (65)	29 (83)	0.3
CPAP/BIPAP	40 (55.5)	18 (49)	22 (63)	0.5
NIV	60 (83.3)	29 (78)	31 (86)	0.3
ETI without NIV	12 (16.7)	8 (22)	4 (11)	0.3
IMV	70 (97.2)	37 (100)	33 (94)	0.5
PEEP (IMV), cm H_2_O, median (IQR)	10 (8–14)	12 (10–15)	10 (6–12)	**0.01**
Scores ECMO initiation, median (IQR)				
SOFA	6 (4–7)	5.5 (4.3–7)	6 (4–7)	0.8
RESP	2 (0–3)	2 (1–3)	1 (-1–2)	**0.01**
Lab values at ECMO initiation				
paO_2_/FIO_2_, ratio, mean (SD)	66 (18)	71 (19)	63 (16)	0.1
pH, mean (SD)	7.31 (0.1)	7.29 (0.1)	7.31 (0.1)	0.6
paCO_2_, mmHg, mean (SD)	63 (20)	65.7 (20)	68.6 (20)	0.5
D-Dimer, µg/L, median (IQR)	1939 (826–3240)	2371 (972–3437)	1707 (556–3029)	**0.05**
Creatinine, mg/dL, mean (SD)	0.70 (0.7)	0.99 (0.7)	0.92 (0.6)	0.7
Bilirubin, mg/dL, mean (SD)	0.44 (0.8)	0.8 (1.1)	0.6 (0.5)	0.5
Platelets, × 10^9^/L, mean (SD)	259 (119)	287 (131)	286 (109)	0.9
Lactate, mmol/L, median (IQR)	2 (1.3–2.6)	1.8 (1.2–2.6)	2.1 (1.5–3.5)	0.1
Days Hospital—ICU, median (IQR)	2 (0—5)	2 (0—5)	2 (0—5)	0.7
Days Hospital—ECMO, median (IQR)	10 (6—16)	9 (5—14)	13 (7—19)	0.2
Days NIV—ICU, median (IQR)	1 (0—2)	1 (0—2)	1 (0—3)	0.5
Days NIV—ETI, median (IQR)	3 (0—6)	2 (0—5)	4 (1—7)	0.07
Days NIV -ECMO, median (IQR)	9 (5—15)	8 (5—13)	9 (6—16)	0.07
Days ETI-ECMO, median (IQR)	4 (2—9)	4 (3—6)	4 (2—11)	0.6

*SOFA* Sepsis-related Organ Failure Assessment, *ICU* Intensive Care Unit, *ECMO* Extracorporeal Membrane Oxygenation, *HFNC* High Flow Nasal Cannula, *CPAP* Continuous Positive Airway Pressure, *BIPAP* Bilevel Positive Airway Pressure, *NIV* Non Invasive Ventilation, *IMV* Invasive Mechanical Ventilation, *PEEP* Positive end Expiratory Pressure, *RESP* Respiratory ECMO Survival Prediction, *ETI* Endotracheal Intubation

Interestingly, RESP score and D-dimer at ECMO initiation were higher among survivors whereas lactate was lower. Times from hospital to VV-ECMO, ICU to VV-ECMO, or intubation to VV-ECMO were all shorter in those patients who survived to day 90 although none of them were statistically significant. Multivariable analysis (Table 2) showed that at VV-ECMO initiation, age, lactate and days from NIV support to VV-ECMO were strongly associated with day-90 mortality.

**Table 2:** Multivariable logistic regression of factors associated with 90-day mortality (considering time lapse between NIV and VV-ECMO)

	OR	95%	*P* Value
Age, yr	1.06	1.01–1.12	**0.02**
PEEP, cm H_2_O	0.93	0.81–1.07	0.28
Days NIV -ECMO	1.03	1.01–1.05	**0.04**
Lactate, mmol/L	1.27	1.11–1.44	**0.01**
D-Dimer, µg/L	1	0.99–1.01	0.49
paO_2_/FIO_2_, ratio	0.99	0.97–1.01	0.19

*PEEP* Positive end Expiratory Pressure, *NIV* Non Invasive Ventilation, *ECMO* Extracorporeal Membrane Oxygenation

To avoid collinearity between days NIV-ECMO and days ETI-ECMO, a different multivariable logistic regression model was performed for the time lapse from ETI to ECMO initiation and 90-day mortality (Table 3). This new model showed a non-significant effect of the time lapse between ETI and VV-ECMO.

**Table 3:** Multivariable logistic regression of factors associated with 90-day mortality considering days from ETI to ECMO

	OR	95%	*P* Value
Age, yr	1.06	1.01–1.12	**0.04**
PEEP, cm H_2_O	0.93	0.81–1.07	0.29
Days ETI -ECMO	1.0	0.95–1.05	**0.69**
Lactate, mmol/L	1.27	1.11–1.44	**0.01**
D-Dimer, µg/L	1	0.99–1.01	0.65
paO_2_/FIO_2_, ratio	0.99	0.97–1.01	0.12

*PEEP* Positive end Expiratory Pressure, *ETI* Endotracheal Intubation, *ECMO* Extracorporeal Membrane Oxygenation

## Discussion

A recent large multicenter study showed an increased mortality in ECMO patients with COVID-19 associated ARDS who had received NIV previous to intubation [16]. In the same line, other observational studies have emphasised the worse prognosis of VV-ECMO patients receiving NIV for too many days previous to endotracheal intubation when compared to those receiving NIV for a shorter period of time [17, 18]. It is important to point out that many of these patients who received NIV during the pandemic are ARDS patients who in other circumstances or in other centers would have been intubated [19, 20]. Furthermore, the majority of scores that predict mortality in VV-ECMO only include the time spent on invasive mechanical ventilation before ECMO (but not on NIV support) [21]. It is interesting to observe how the RESP score which is one of the scales most used and recommended by the ELSO (Extracorporeal Live Support Organization) to predict mortality before the start of ECMO, includes the number of days that patients have been on invasive mechanical ventilation but does not consider the time lapse from previous NIV to intubation [22–24].

In our cohort of VV-ECMO patients with COVID-19 associated ARDS, the time lapse between NIV support and commencing VV-ECMO (together with age and lactate) seems a better predictor when evaluating survival than the time between intubation and VV-ECMO. Age and lactate are clearly related to mortality when VV-ECMO is initiated as in the majority of critically ill patients who present any kind of organ dysfunction [16, 21]. In fact, both age and lactate are included in the majority of the risk-assessment severity scores used for any kind of ECMO support [25, 26]. Nevertheless, due to the improvement of organ transplant programs (including lung transplant), the better quality of life of older people, and due to other more complex social demands, age keeps being a time changing criteria and nowadays treatment indications are being extended to groups of age that not long ago would have been excluded. This includes also VV-ECMO indications that in the past were clearly age-limited but nowadays have to be patient-personalised because some of these “older” patients might even be candidates for lung transplant or might have excellent qualities of life with no other comorbidities [27].

Although in our small cohort of patients the time lapse from ETI to VV-ECMO was not significant in terms of mortality it is one of the most employed criteria when evaluating the mortality risk of the technique as those patients initiated more than 7 days after ETI have proved to have worse outcomes than those initiated within the first week. This is probably related to a more advanced stage of ARDS in those patients with a delayed initiation of VV-ECMO when compared to those with an early initiation of VV-ECMO. However, this phenomenon is common to other forms of mechanical organ support in critically ill patients such as renal replacement therapy or invasive mechanical ventilation, where a delayed initiation strategy is associated with a worse outcome when organ support is finally required, but also with a much better outcome when patients finally do not require organ support [28].

The -timing of the intervention- issue has been also explored in ECMO patients for prone positioning [29]. Even in this field, when applied in a late phase, these interventions seem to be ineffective. An early identification of all those patients who will finally require organ support in a delayed and deleterious period is probably one of the most compelling brain exercises that still exist in critical care medicine together with the appropriate quantity of fluids to be used in patients with shock.

An important confounding variable in the assessment of time from NIV initiation to VV-ECMO is the duration of invasive ventilation that somehow makes our hypothesis seem overly simplistic. This is an important limitation of our study design. In our study, no association was found between the use of NIV previous to VV-ECMO and mortality, although the small size of the population is another important limitation. The variables selected for the multivariable logistic regression model were based on the statistical significance and not on a clinically meaningful method due to the limited number of events (35 deaths). This should be considered also as a limitation of our study. On the other hand, the lack of information on the pressures applied to these patients and the absence of cross-sectional protocols does not allow the identification of patients who have been treated with high parameters perhaps comparable to those of intubated patients. It seems reasonable that patients with severe stages of ARDS may require higher pressures of NIV (CPAP or BIPAP) that with HFNC cannot be achieved. Some trials performed during the COVID pandemics have reported results in this direction revealing that higher pressures with NIV could be associated with lower rates of ETI or even mortality [30, 31]. A confounding variable in most studies could be the failure to differentiate patients who received HFNC from those who received BiPAP or CPAP, understanding that these last patients who require higher pressures are those who present severe ARDS [30, 31].

## Conclusions

We suggest, based on our findings, that the time spent between NIV support and initiation of VV-ECMO should be considered and evaluated as an interesting clinical parameter to be included in the mortality prediction scores for VV-ECMO although further and larger studies are needed to confirm our findings [32].

## Data Availability

All data generated or analysed during this study are included in this published article [and its supplementary information files].

## Abbreviations

VV-ECMO: Veno venous extracorporeal membrane oxygenator
COVID-19: Coronavirus disease 2019
NIV: Non-invasive ventilation
HFNC: High Flow nasal cannula
BiPAP: Bi-level positive airway pressure
CPAP: Continuous positive airway pressure
NIPPV: Non-invasive positive pressure ventilation
ARDS: Acute respiratory distress syndrome
ARF: Acute respiratory failure
ICU: Intensive care unit
P-SILI: Patient self-inflicted lung injury
SOFA: Sequential Organ Failure Assessment
RESP: Respiratory ECMO survival prediction
IQR: Interquartile range
CI: Confidence interval
OR: Odds ratio
PEEP: Positive end expiratory pressure
ETI: Endotracheal intubation
SEMICYUC: Spanish critical care society
ELSO: Extracorporeal Life Support Organization
